# Bed Bug (*Cimex lectularius* L.) Population Composition as Determined by Baited Traps

**DOI:** 10.3390/insects3020442

**Published:** 2012-04-30

**Authors:** Elizabeth J. Schaafsma, Samuel D. Hapke, Michael G. Banfield

**Affiliations:** SpringStar Inc., P.O. Box 2622, Woodinville, WA 98072, USA; E-Mails: shapke@wsu.edu (S.D.H); mike@springstar.net (M.G.B.)

**Keywords:** *Cimex lectularius*, bed bug, trap, monitor, carbon dioxide, kairomone, population sampling technique

## Abstract

Two established field populations of bed bugs were sampled using host-mimicking traps baited with a combination of CO_2_, heat and a synthetic kairomone. The proportion of first instar nymphs (between 52% and 78% of all captured insects) was significantly higher than reported in previous studies, which had employed different sampling methods. The proportion of adults was correspondingly much lower than previously reported, between 5% and 7% of total capture. As many as 120 bed bugs were captured in a single trap in one night; the variation in catches between sampling locations within the same room and between days at the same location indicates that multiple nights of trapping may be required to obtain an accurate representation of population structure.

## 1. Introduction

The common bed bug, *Cimex lectularius* L, is a cosmopolitan pest of human habitation, with documented infestations dating back to ancient Greece [1] and Pharaonic Egypt [2]. With the advent of DDT and other synthetic insecticides during WWII, bed bugs became somewhat of a rarity in industrialized countries [1,3]. As bed bugs are not known to transmit human disease [4], scientific interest in them waned along with their prevalence [5]. Since the 1990s, however, many countries including the United States have experienced exponential increases in bed bug populations [1,5]. This widespread resurgence is currently attributed to a combination of insecticide resistance, changes in control strategies for other urban pests, and an increase in travel both within and between countries [6].

Bed bugs are a difficult pest to study in their native habitats, as a result of their small size and cryptic habits. Their bodies, which are 1–7 mm long [7] and dorso-ventrally flattened [7], allow them to hide in narrow cracks. They are generally nocturnal, with maximum activity between midnight and 6:00 am [8,9]. Under ideal laboratory conditions, bed bugs will go through five nymphal stages [7], each lasting 4–8 days [3] before a final molt to an adult stage. Depending on the strain of bed bug, their level of resistance to pesticides [3,10], activity level [9], the ambient temperature and humidity [11], and host availability, adult bed bugs may live for anywhere from three months up to four years [3]. Given the breadth of factors documented to affect stage-specific survival in the laboratory, the number of adults seen or captured in the field may not be reliably correlated with the size of the total population [3,5,12]. The proportion of adult bed bugs within a discrete population has been reported as anywhere from 6% [12] to 68% [13] of observed life stages. A controlled laboratory study calculated that the stable age distribution of one pyrethroid-resistant strain would include approximately 19% adults [3]. 

While stable age distributions are unlikely to be encountered in nature [3], comparisons between them and observed population metrics can suggest the presence of factors influencing field populations, such as environmental pressures, migration rates, and the age of an infestation. The ecology and population dynamics of *C. lectularius* are perhaps the least studied areas of bed bug biology in recent years [5]; yet, an understanding of these dynamics may aid in the development of monitoring and control measures. Few ecological field studies have been documented since Usinger’s monograph [7], and while the older studies are useful, it is unlikely that the bed bug populations of over 70 years ago are identical to the field strains that are encountered today [3].

A variety of techniques and devices may be used to detect and survey bed bug populations [14]. Visual inspections are commonly used to detect bed bugs, but are time and labor-intensive [14], and even trained individuals can miss large numbers of insects [15]. Bed bug-sniffing dogs are becoming an increasingly common survey method, especially for detecting low-level infestations, but they are expensive to train and employ, are not available in all areas, and their accuracy can vary widely between dog-and-handler teams [14]. Recent studies [14,16] have demonstrated the potential for trapping as a viable alternative to visual or canine inspections in confirming the presence and size of an infestation. Wang *et al*. [14] showed that certain traps can detect up to 100% of infestations previously identified by visual inspection.

Additionally, traps baited with host-mimicking attractants have the potential to be used to study the field ecology of bed bugs: after emerging from eggs, bed bugs in all developmental stages are obligate blood feeders and thus must search for a host. Traps with carbon dioxide (a primary host-produced attractant) have been demonstrated to catch all motile life stages of bed bugs [16]. However, there are currently no published studies investigating the accuracy or bias of any host-based traps for population sampling in the laboratory, nor have any attempts been made to determine their usefulness in studying natural infestations. In this study, we present the results of sampling two established bed bug infestations with host-mimicking traps and compare them to published population data to examine the utility of this population sampling technique.

## 2. Methods

### 2.1. Trap Description

The trapping device used for this study was the First Response Bed Bug Monitor (SpringStar Inc., Woodinville, WA, USA), which uses a combination of CO_2_, heat and a synthetic kairomone lure to attract bed bugs. The kairomone lure is a proprietary blend of chemicals designed to mimic a sleeping human host. Exact kairomone lure components and concentrations were not disclosed by the manufacturer; however, the lure and trap are commercially available

**Figure 1:**
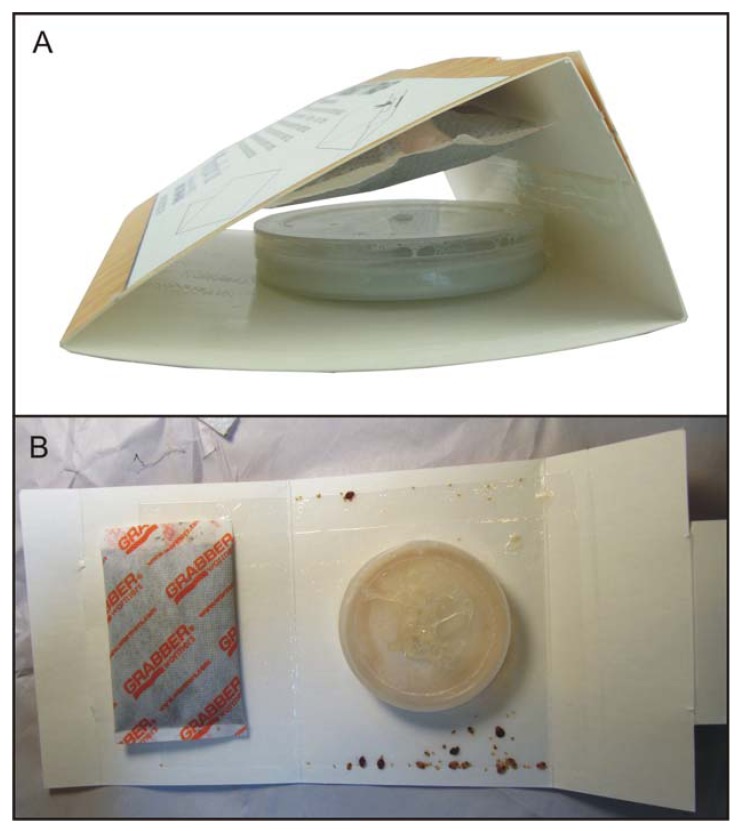
(**a**) Monitor, assembled; (**b**) Monitor after 24 hours in the field, unfolded for counting and identification.

The First Response Bed Bug Monitor (hereafter referred to as the Monitor, Figure 1A) consists of: a white adhesive-coated paperboard, folded into a triangle, which can be unfolded for counting and identification (Figure 1B); a 25 mL plastic fermentation container; a disposable heating pad, similar to a hand-warmer; and a 1 mL vial containing 150 µL of the kairomone blend. The CO_2_ container is activated by adding ~13 mL of water. The heat source is activated by opening the foil wrapper. The kairomone blend is then added directly to the warmer to increase the release rate. Each Monitor is intended for a single-use: the fermentation reaction produces 40–50 mL/hr of CO_2_ for at least 8 hours at room temperature (18–22 °C), as measured by water displacement; and the heating pad was measured, using a handheld infrared thermometer, as maintaining a surface temperature greater than or equal to average human skin temperature [17] for 8–12 hours.

### 2.2. Study Site 1: Unoccupied Room in Downtown Homeless Shelter with No Furniture

The first study site was a 10’ × 20’ room within a three-story homeless shelter in downtown Seattle, WA. Because of budget constraints at the facility, only limited treatments had been performed over the last several years in an unsuccessful attempt to keep the bed bug population at tolerable levels. Sporadic steaming and/or baseboard spraying with Phantom® (chlorfenapyr; BASF Corporation) had occurred a maximum of two to three times per year. The room chosen as the study site had one of the highest population densities in the facility as reported by staff members and guests; additionally, it had received the fewest treatments of all infested rooms. A brief visual inspection found at least 500 bed bugs (adults and nymphs) in the carpet, trim, wallboards, and windowsill up to five feet from the floor. During the day, the room was commonly used for small meetings, while at night the room was one of several housing sleeping guests. There was no permanent furniture in the room; residents would sleep on thin mats spread directly on the floor which were collected each morning. During the course of the study, residents did not sleep in the room, although they did sleep in the hallway directly outside the room. In addition, the room was generally left locked and unused during the study duration.

After the visual assessment, one Monitor was placed in each of one (1 night), three (6 nights) or four (7 nights) corners of the room for 14 nights between 26 April and 24 May, 2011. Monitors were labeled with the date of placement and location, and then placed along the baseboard trim, within two feet of the corner. All Monitors were placed between 2:00 pm and 11:00 pm and retrieved around 24 hours later. The collected devices were bagged in re-closable plastic bags and frozen at −20 °C for at least 24 hours. After freezing, the Monitors were disassembled, and trapped bed bugs were counted and classified by development stage and, for adults, sex.

### 2.3. Study Site 2: Occupied 2-Bedroom Duplex with Furniture

The second study site was an occupied, two-bedroom duplex outside of Seattle, WA. The residents reported a bed bug infestation on 19 April 2011. During the initial inspection, the residents further stated that the unit had been infested since they moved in (ca. two years prior) and that no treatments, professional or otherwise, had been attempted or undertaken in that time. A visual survey with flashlights established that the infestation was mainly confined to the two bedrooms; aspirated samples from the carpets suggested an estimated population size in the thousands in one of the bedrooms.

Two residents slept on mattresses placed directly on the floor in one of two corners of each bedroom, with a fifth resident sleeping on a couch in a separate room. The study was conducted in the two bedrooms. Prior to trap placement, all mattresses were moved off the floor onto cots, and liquid-filled cups were placed as barriers underneath each bed leg.

One Monitor was placed per bed per night over 12 nights between 7 May and 7 June 2011. Traps were placed under the bed and against the baseboards, nearest to the greatest observed population densities. All Monitors were labeled with date of placement and bed number, placed between 9:00 pm and 11:00 pm, and then retrieved around 24 hours later. The collected devices were bagged in reclosable plastic bags and frozen at −20 °C for at least 24 hours. Then the Monitors were disassembled and trapped bed bugs were counted and classified by development stage and, for adults, sex.

### 2.4. Data Analysis

All bed bug counting was performed under bright light. Bed bugs were identified to developmental stage and sex using a photographic key created by S. Doggett [18]. Adult bed bugs were examined under a dissecting microscope (6.7×–40× magnification) to determine gender. Fully replete insects were counted but usually could not be classified to instar or gender because of the distention of body size and shape resulting from feeding.

To compare average trap catches between two time periods (Site 1) or two rooms (Site 2), the independent samples t-test with Levene’s test for equality of variances was used [19]. When comparing average trap catches between more than two groups (e.g., all four corners at Site 1), a log-transformed, univariate analysis of variance [19] was used to analyze the data. The binomial test [19] was used to compare ratios of adult male and female bed bugs to the expected 50:50 ratio [5,12,20,21]. Proportions of life stages between weeks or locations were compared using a goodness-of-fit contingency table [19]. Changes in proportions of adults to nymphs between weeks or locations were compared using Fisher’s exact test [19]. Changes in the average number of insects per day over time were analyzed using linear regression [19]; the log-transformed averages were used for Study Site 1, to correct for outliers and heterogeneity of variance. Statistical analyses were performed using SPSS (SPSS 19, IBM, 2010).

## 3. Results

### 3.1. Study Site 1: Unoccupied, Unfurnished Room in Homeless Shelter

A total of 19 traps were placed over seven consecutive nights from 26 April to 2 May, and an additional 28 traps were placed over seven nights between 14 May and 24 May. The total number of bedbugs captured was 699 during the first time period and 1037 during the second. The average (±SE) number of bed bugs caught per trap was not significantly different between the two time periods (36.8 ± 7.4 *vs*. 37.0 ± 6.3, *p* = 0.980). The average number of bedbugs collected on each trapping date increased significantly during the first seven-day trapping period (R^2^ = 0.825, df = 6, *p* = 0.005). Trap catches fluctuated, with a non-significant overall decline, during the second seven-day trapping period (R^2^ = 0.372, df = 6, *p* = 0.146). Even with first-instar numbers removed, no significant trend in trap catches was observed during the second week (R^2^ = 0.423, df = 6, *p* = 0.114), indicating that the fluctuation was not due solely to mass hatching events. The average number of bedbugs caught per trap increased from 12 on 26 April to 55 on 2 May. A maximum of 293 total bed bugs were trapped in one night on 18 May. 

A great deal of variation was observed in the number of bed bugs caught in any one trap; the total catch in a single trap ranged from 2 insects in a night up to 120. Some of the variation was due to location, as traps in some corners caught significantly more bed bugs than traps in other corners on the same night (F_3,43_ = 13.332, *p* < 0.001). Some variation was also temporal; up to a ten-fold difference was observed in total trap catches from one night to the next in a single location (e.g., 24 total bed bugs in Corner D on 14 May, 120 total bed bugs in the same corner on 15 May). However, trapping was not performed over enough consecutive days to determine if there was a consistent pattern to the daily variation in catch numbers.

Significant differences were noticed in the proportion of each life stage captured between the first week of trapping and the second (χ^2^ = 246.8, df = 5, *p* < 0.001) (Table 1). Proportionally more first instar nymphs were captured during the second trapping period than the first, and proportionally less of all other nymphal stages, especially second instar nymphs. However, the ratio of adults to nymphs did not change significantly between the two weeks (*p* = 0.330). Additionally, the male:female ratio of trapped adults switched between the two trapping periods, from female-biased (*p* = 0.007) to male-biased (*p* = 0.009).

**Table 1:** Proportion of life stages trapped during two separate weeks at Study Site 1.

Location	Date Range	# of Traps	Nymphs					Adults	Total Insects Trapped
			N1	N2	N3	N4	N5		
Site 1, week 1	4/26–5/2	19	52%	27%	7%	6%	3%	6%	699
Site 1, week 2	5/14–5/24	28	78%	7%	3%	3%	3%	5%	1037

### 3.2. Study Site 2: Occupied Duplex with Furniture

A total of 44 traps were placed in the two bedrooms over 11 non-consecutive nights between 8 May and 7 June. During that time period, 696 bed bugs were trapped. The back bedroom was visibly more infested; 79% of all bed bugs were trapped in there. Individual trap catches (Mean ± SE) were also higher in the back bedroom (27.0 ± 3.1 bed bugs/trap) than the front (4.6 ± 0.7 bed bugs/trap). Despite the difference in total number of insects caught, the proportion of each feeding stage caught in the two rooms was not significantly different (χ^2^ = 4.5, df = 5, *p* = 0.478; Table 2). The male-to-female ratio was also essentially even in both rooms (Back: *p* = 0.711. Front: *p* > 0.999).

**Table 2:** Proportion of life stages trapped in two bedrooms at Study Site 2.

Location	Date Range	# of Traps	Nymphs					Adults	Total Insects Trapped
			N1	N2	N3	N4	N5		
Site 2, back room	5/8–6/7	22	73%	13%	4%	2%	2%	5%	594
Site 2, front room	5/8–6/7	22	72%	9%	6%	5%	2%	7%	102

Due to difficulties with access to the residence, trapping could not be performed on consecutive nights with any regularity. Thus the daily variation within a location could not be tracked. However, during the month-long study period, a significant decline (R^2^ = 0.453, df = 10, *p* = 0.023) was observed in the total number of bed bugs caught per night in the back room; there was no significant change in the total number of bugs caught per night in the front room (R^2^ = 0.086, df = 10, *p* = 0.382).

## 4. Discussion

This study demonstrated that in certain established bed bug infestations there may be more early-stage nymphs, especially first instar nymphs, than previously reported. In all locations and time periods, between 52% and 78% of trapped bed bugs were first instar nymphs. Additionally, adult bed bugs comprised only 5–7% of all trapped insects. These results are especially interesting when compared to previously reported distributions of populations from a variety of field and laboratory conditions.

**Figure 2:**
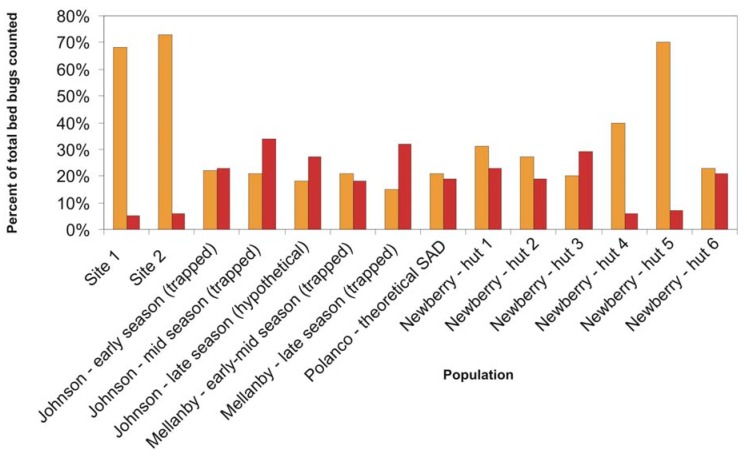
Proportion of feeding stages consisting of first instar nymphs (orange) and adults (red) in bed bug populations described in this study and in select studies from the literature.

Previous population studies of field and laboratory populations have reported a wide range of population composition values (Figure 2). Reinhardt and Siva-Jothy [5] mentioned that adults form approximately one third of cimicid populations. Johnson [20] found that adults comprised 45–100% of “overwintered” populations, that is, populations which had spent the entire winter fasting in unheated bedrooms. Populations that had not experienced annual winter die-offs or had several months to reproduce were comprised of 15–22% first instar nymphs and 7–16% adults [20]. Mellanby, working in a heated, infested animal room, captured 15–22% first instar nymphs and 18–32% adults in un-baited pitfall traps [9]. Polanco *et al*. [3] developed a life table for one lab-reared population of pyrethroid resistant bed bugs and calculated that adults would represent about 19% and first instar nymphs would represent 21% of feeding stages in the stable age distribution (SAD). Newberry and Jansen [12], in their studies of *C. lectularius* populations in African huts, found that adults ranged from 5.6–30.0% of feeding stages, and first instar nymphs made up anywhere from 18.8% to 69.8% of the mobile population. Their labor-intensive sampling method, which included fumigating entire huts after lining the floors with white sheets, likely provides a more accurate portrait of the younger instars than visual inspection. In lieu of destructive sampling of established infestations (*i.e*., ripping apart a building and counting every bed bug inside), the Newberry and Jansen method could represent the closest approximation of an entire bed bug population in the field. 

The population compositions we determined via trapping are within the range of previously reported populations, albeit highly skewed towards first instar nymphs, and with a much lower adult proportion than generally reported. If our trapped population closely approximates the total population in the locations studied, then these findings have significant implications for bed bug management strategies. The high proportion of early-stage bed bugs could mean that many young insects are being missed during post-treatment surveys, leading to premature termination and ultimate failure of treatment. However, it is also possible that the Monitor used in this study differentially attracts 1st instar nymphs and thus overestimates their percentage of the population. Further testing in a controlled setting is needed to explore that hypothesis. Alternatively, the adhesive could be less efficient at catching or holding larger nymphs and adults. As we observed no escapees or partially trapped insects during trap collection, and no escapees from bagged traps which were not frozen immediately after collection, differential capture efficiency was not considered likely. 

There are currently no published studies comparing the accuracy of various methods of population sampling for bed bugs, either inside or outside the laboratory. The closest analogue with published comparative trapping studies would be cockroaches. Although not a parasitic pest, cockroaches do share several important ecological characteristics with bed bugs, including: nocturnal activity; flattened bodies, which allow them to hide in cryptic structural habitats; widespread distribution in human environments; aggregation close to food; populations consisting of intermingled nymphs and adults [22]; and high intrinsic rate of reproduction [3]. Reierson and Rust [23] found that both baited trapping and pyrethroid flushing consistently detected German cockroaches in more apartments than visual counting alone. However, nymphs made up a much higher proportion of trapped roaches than were observed with either of the other two survey methods. The authors concluded that the difference “could be due to our inability to locate small nymphs that were knocked down or escaped into hidden harborage areas” and that trapping “probably revealed numbers closer to actual field populations” [23]. Owens and Bennett [24] investigated the precision of flushing, visual counting, and several different baited traps in the laboratory, and found that one type of baited trap had the least sampling bias of all methods studied, despite sampling the smallest portion of the population. 

Although focused on cockroaches, both studies demonstrate an adult bias in visual counting methods resulting from the difficulty of seeing the smaller nymphs. Given the extremely small size (1–3 mm) [7] of early-instar bed bug nymphs, an adult bias similar to that documented in cockroach sampling could impact the efficacy and accuracy of visual inspection for bed bugs. Newberry and Jansen [12] commented on this phenomenon, noting that manual collection of knocked-down bed bugs missed most of the early stages; thus, to ensure accurate sampling of all *C. lectularius* stages, any insects collected via knockdown had to be carefully swept up and examined under a microscope. As demonstrated in cockroach sampling studies, baited traps have the potential to be a valuable tool for sampling cryptic anthropophilic pests without the biases inherent to visual inspection. Laboratory testing and/or more field testing is still needed, however, to determine the accuracy or bias of the available baited bed bug traps.

In addition to the unexpectedly nymph-biased composition of the populations, significant changes in population composition and gender ratios were observed over the duration of the experiment. However, a majority of these changes were noted at only one of the two locations studied. Both study sites housed long-established bed bug infestations, in areas where the residents slept on mattresses directly on the floor. Consequently, the main harborages at sites were in and around the walls instead of in bed frames or box springs. It was expected that the populations in the two locations would be similar and would change in similar ways over time. It is possible that the sudden absence of hosts at Site 1 *vs*. the continued (albeit isolated) presence of the hosts at Site 2 may have had an effect on the difference in changes over time. Fewer bed bugs were caught per trap in the inhabited rooms, but whether that was due to population size or competition from the more attractive hosts could not be determined. The similarities in population composition between the two study sites suggest that the presence of a host may not confound insect response to the traps, but further study of the effect of host presence on the accuracy of population survey data via trapping may be warranted. 

This present investigation also provides some baseline information on the utility of baited trapping as a method for surveying the population structures of bed bugs. Based on the results of this study, baited traps are a promising alternative to other methods for surveying populations of bed bugs. Baited traps can be set and retrieved by relatively unskilled personnel, with final counting and analysis performed in the laboratory by one or a few trained individuals. This would allow limited resources to cover a much broader survey area. Additionally, less time could be spent in the field and destructive or disruptive sampling (*i.e*., disassembling furniture to check all seams and cracks) would not be required, nor would results be dependent on the variability of individual efforts. Both advantages would also be less disruptive to residents, possibly expanding the number of areas accessible for such studies. 

There are some drawbacks to baited trapping. Host-mimicking traps target all mobile stages, but cannot detect non-mobile stages (e.g., eggs). Additionally, it is unknown from what maximal distance bed bugs can detect host cues, meaning that either trained personnel are still required to do initial surveys to determine the main infestation sites and best places to set the traps, or traps numbers should be increased significantly. Finally, the traps used in this study run for only 8–10 hours after setup, which required the researchers to assemble new traps every night, and to set them up in the evening, after standard working hours. It has been estimated that bed bugs feed every 3–8 days [5,9,21]. Adult females may also synchronize their feeding [21]. Thus multiple nights of trapping are likely necessary to obtain a representative sample of the population. A trap that is consistently attractive for at least a week could potentially allow a researcher to obtain sufficient data with a single device.

Previous researchers [3] have noted the need for sampling methods that can account for the abundance of the small, early immature stages of bed bugs. A host-mimicking trap (such as the one used in this study) baited with CO_2_, heat and host kairomones may be one answer to this need. It will be necessary to determine the biases inherent in the trapping method before it can be relied upon as a stand-alone sampling tool. An accurate, fast, and relatively simple population sampling technique would be an important step towards control of this rapidly resurging pest.

## 5. Conclusions

The only available studies evaluating the accuracy of traps for sampling populations of residential arthropod pests were focused on cockroaches. This study provides a preliminary evaluation of baited traps for population sampling of bed bugs. Bed bug populations in two heavily infested, untreated locations were sampled using traps baited with host-mimicking cues. In both populations, the proportion of the population represented by early stage nymphs (specifically first instar nymphs) was higher than generally reported in previous studies using different sampling methods. Further research is needed to illuminate the reasons underlying the differences between the population compositions observed here and in previous studies.
